# Free-living bacterial communities associated with tubeworm (*Ridgeia piscesae*) aggregations in contrasting diffuse flow hydrothermal vent habitats at the Main Endeavour Field, Juan de Fuca Ridge

**DOI:** 10.1002/mbo3.70

**Published:** 2013-02-09

**Authors:** Nathalie L Forget, S Kim Juniper

**Affiliations:** 1 Department of Biology, University of Victoria3800 Finnerty Rd, Victoria, British Columbia, Canada, V8P 5C2; 2 School of Earth and Ocean Sciences, University of Victoria3800 Finnerty Rd, Victoria, British Columbia, Canada, V8P 5C2

**Keywords:** 454 tag sequencing, bacterial diversity, hydrothermal vents, microbial ecology, pyrosequencing, *Ridgeia piscesae*, SSU rRNA gene

## Abstract

We systematically studied free-living bacterial diversity within aggregations of the vestimentiferan tubeworm *Ridgeia piscesae* sampled from two contrasting flow regimes (High Flow and Low Flow) in the Endeavour Hydrothermal Vents Marine Protected Area (MPA) on the Juan de Fuca Ridge (Northeast Pacific). Eight samples of particulate detritus were recovered from paired tubeworm grabs from four vent sites. Most sequences (454 tag and Sanger methods) were affiliated to the *Epsilonproteobacteria*, and the sulfur-oxidizing genus *Sulfurovum* was dominant in all samples. *Gammaproteobacteria* were also detected, mainly in Low Flow sequence libraries, and were affiliated with known methanotrophs and decomposers. The cooccurrence of sulfur reducers from the *Deltaproteobacteria* and the *Epsilonproteobacteria* suggests internal sulfur cycling within these habitats. Other phyla detected included *Bacteroidetes*, *Actinobacteria*, *Chloroflexi*, *Firmicutes, Planctomycetes*, *Verrucomicrobia,* and *Deinococcus–Thermus*. Statistically significant relationships between sequence library composition and habitat type suggest a predictable pattern for High Flow and Low Flow environments. Most sequences significantly more represented in High Flow libraries were related to sulfur and hydrogen oxidizers, while mainly heterotrophic groups were more represented in Low Flow libraries. Differences in temperature, available energy for metabolism, and stability between High Flow and Low Flow habitats potentially explain their distinct bacterial communities.

## Introduction

Shortly after the discovery of hydrothermal vents and their dense, exotic fauna in 1977 (Lonsdale 1977), the role of chemoautotrophic bacteria and archaea in the production of new organic matter was recognized as the basis of food webs in these ecosystems (Corliss et al. 1979; Jannasch and Wirsen 1979; Karl et al. 1980). The first surveys of free-living vent microbial communities, based on morphological observations, revealed the presence of different types of sulfur-oxidizing organisms as well as possible methylotrophic and nitrifying bacteria (Jannasch and Wirsen 1981). Subsequent culture-based studies of hydrothermal vent microorganisms focused mainly on specific groups, such as sulfur bacteria (Ruby et al. 1981, 1987; Felbeck and Somero 1982; Wirsen et al. 1986) and thermophilic organisms (Baross and Deming 1983; Jones et al. 1983; Belkin et al. 1986; Jannasch et al. 1988). Eventually, the development of molecular techniques overcame the limitations of culture enrichments, and molecular tools are now permitting a more thorough portrayal of free-living microbial diversity at hydrothermal vents in a broad range of geological settings.

Molecular surveys of prokaryotic diversity at vents are still relatively few, but some general patterns are emerging. For example, many studies have found the class *Epsilonproteobacteria* to dominate free-living microbial communities associated with sulfide chimneys (Pagé et al. 2004; Kormas et al. 2006; Zhou et al. 2009), microbial mats (Moyer et al. 1995; Longnecker and Reysenbach 2001; Moussard et al. 2006), hydrothermal fluids (Huber et al. 2003, 2010), and vent fauna (Polz and Cavanaugh 1995; Alain et al. 2002, 2004; Lopez-Garcia et al. 2002; Pagé et al. 2004; Petersen et al. 2010). Most studies are also finding that phylogenetic diversity at vents tends to be higher than in other marine environments (Forget et al. 2010) and it is beginning to appear that this diversity can be quite dynamic, varying considerably between vents within a same field (Nakagawa et al. 2005c; Davis and Moyer 2008; Huber et al. 2010), at individual vents under different environmental conditions (Reysenbach et al. 2000; Byrne et al. 2009), and between different zones of sulfide edifices (Schrenk et al. 2003; Kormas et al. 2006; Pagé et al. 2008). Faunal aggregations at vents provide physical habitat for free-living microorganisms and a few studies have attempted to characterize the free-living microbial communities associated with different faunal communities (Prieur et al. 1990; Polz and Cavanaugh 1995; Campbell et al. 2001; Zbinden et al. 2008). Compositional changes in macrofaunal communities have been investigated within a framework of identifying successional processes driven by habitat change (Fustec et al. 1987; Hessler et al. 1988; Tunnicliffe and Juniper 1990; Sarrazin et al. 1997, 1999, 2002; Tunnicliffe et al. 1997; Sarrazin and Juniper 1999; Cuvelier et al. 2009, 2011), but little is known of how the composition and diversity of associated free-living microbial communities might be related to habitat dynamics and faunal succession. One example is the comparison by Pagé et al. (2004) of the microbial diversity associated with the “sulfide worm” *Paralvinella sulfincola* with a similar study from Alain et al. (2002) involving the “palm worm” *P. palmiformi*s at Axial volcano, Juan du Fuca Ridge. These two species of alvinellid polychaetes represent the first and second colonizers of newly formed chimney surfaces of Northeast Pacific vents, respectively (Sarrazin et al. 1997). The major taxonomic bacterial groups, dominated by *Epsilonproteobacteria*, were comparable between the two studies, but thermophilic bacterial groups and Archaea were also detected in association with the first colonizer *P. sulfincola* (Pagé et al. 2004). However, the lack of replication limited the interpretation of these results.

We present here a systematic study of microbial diversity in two contrasting, previously described habitats of the vestimentiferan tubeworm *Ridgeia piscesae* located in the Endeavour Hydrothermal Vents Marine Protected Area (MPA), Juan de Fuca Ridge*. R. piscesae* represents the major foundation species of the MPA, providing food, colonization surfaces, and shelter to smaller species such as gastropods and other polychaetes. The first habitat corresponds to the Assemblage V-High Flow faunal community described in Sarrazin and Juniper (1999) and Sarrazin et al. (1999, 2002), which is dominated by the short-fat morphotype of *R. piscesae* exposed to vigorous fluid flow, hydrogen sulfide concentrations around 40 μmol/L, and temperatures up to 41.9°C. High faunal density and biomass, but low species diversity characterize this “High Flow” habitat. Other macrofaunal species present include the alvinellid polychaetes *P. sulfincola* and *P. palmiformis*, as well as a few polynoid polychaete species. The second habitat, which will be referred to as “Low Flow”, is dominated by widespread aggregations of the long-skinny morphotype of *R. piscesae* in areas of weaker hydrothermal discharge. At the tubeworm plume level, the temperature is usually just above that of ambient seawater temperature, around 2°C, and sulfide concentrations are extremely low (<0.1 μmol/L) (Urcuyo et al. 1998, 2003). This study constitutes the first survey of free-living bacterial diversity associated with this vestimentiferan.

Our comparative study, which combined Sanger sequencing and 454 tag sequencing, was based on four replicates for each of the two habitats (Table 1), allowing for thorough statistical analyses and a better understanding of the distinguishing features of their respective microbial communities.

**Table 1:** Description and location of sampling sites

Sample	Tubeworm morphotype	Flow regime	Vent site	Latitude	Longitude	Site description	Depth (m)	Max. temp. at plume (°C)
HF8SMb	Short-fat	High	Smoke & Mirrors	47°56.879′N	129°5.912′W	Lots of shimmering, proximity to black smoker	2180.6	10.0
LF8SMb	Long-skinny	Low	Smoke & Mirrors	47°56.880′N	129°5.911′W	Little shimmering, no black smoker visible	2180.5	5.0
HF8GH1b	Short-fat	High	Grotto (Hotspot 1)	47°56.948′N	129°5.896′W	Black smoke all around	2188.2	30.0
LF8GH1b	Long-skinny	Low	Grotto (Hotspot 1)	47°56.955′N	129°5.900′W	No shimmering visible	2187.9	3.6
HG8GH2b	Short-fat	High	Grotto (Hotspot 2)	47°56.951′N	129°5.899′W	Black smoke all around	2188.1	30.0
LF8GH2b	Long-skinny	Low	Grotto (Hotspot 2)	47°56.944′N	129°5.899′W	Little shimmering, no black smoker visible	2190.4	11.4
HF8CBb	Short-fat	High	Clambed	47°57.778′N	129°5.490′W	Lots of shimmering, proximity to black smoker	2188.3	27.0
LF8CBb	Long-skinny	Low	Clambed	47°57.770′N	129°5.490′W	No shimmering visible	2191.3	2.4

## Experimental Procedures

### Site description and sample collection

**Figure 1:**
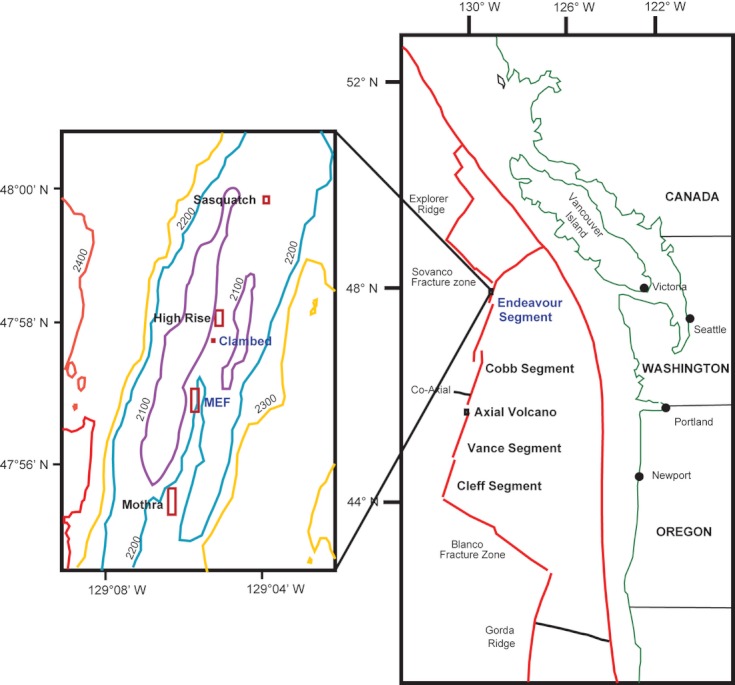
Map of the northeast Pacific (modified from Bourbonnais et al. 2012) showing the location of the Endeavour Segment on the Juan de Fuca Ridge, as well as the major vent fields of this segment. The sample collection sites are in blue. (MEF = Main Endeavour vent field).

**Figure 2:**
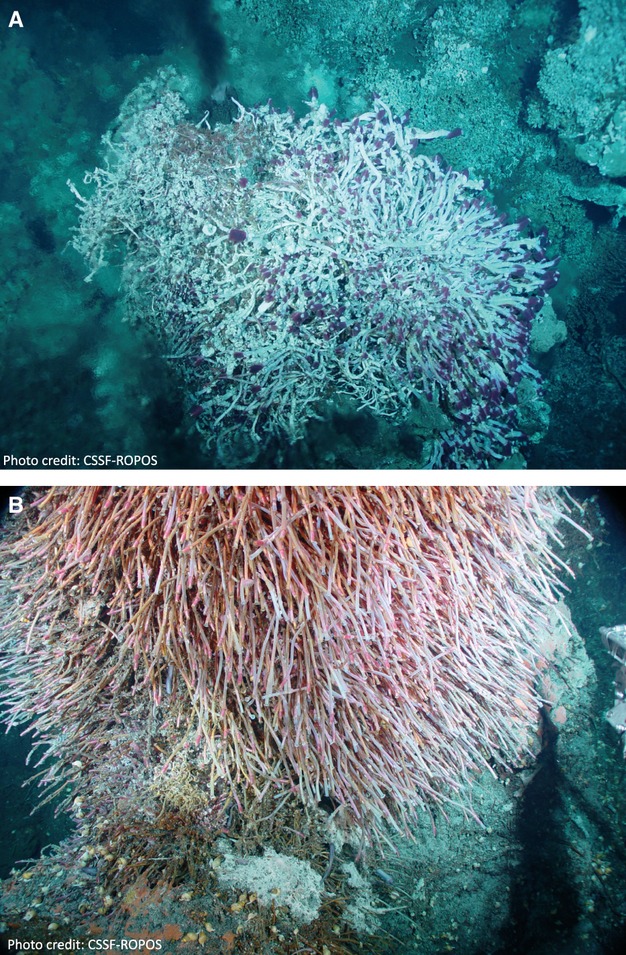
Examples of typical sampling sites. (A) High Flow environment inhabited by the short-fat phenotype of *Ridgeia piscesae*. The shimmering indicates the presence of hydrothermal fluid venting through the aggregation tubeworms. (Note their white tube and bright-red healthy-looking branchial plume.) (B) Low Flow envi-ronment inhabited by the long-skinny phenotype of *R. piscesae*. No shimmering is visible. (Note their brown-orange color of the tube and the reduced branchial plume.)

The samples used in this study were collected from three different sites in the Main Endeavour vent field, that is, the Smoke & Mirrors (S&M) edifice, and Hotspot 1 and Hotspot 2 on the Grotto edifice; and from a single site in the Clam Bed vent field, during a 2008 research expedition on board the Canadian Coast Guard Ship *John P. Tully*. At each site (Table 1 and Fig. 1), paired grab samples of the most contrasting morphotypes of the tubeworm *R. piscesae*, known as the “short-fat” and the “long-skinny” morphotypes, were collected from their respective habitats within a distance of 10 m using the remotely-operated vehicle (ROV) remotely operated platform for ocean sciences (ROPOS) and placed in separate, closing bioboxes. Aggregations of the short-fat morphotype were typically found near a black smoker in relatively high temperature and vigorous diffuse fluid flow areas (Fig. 2A). The long-skinny morphotype, which is more common and widely spread, was collected in regions of low-temperature diffuse hydrothermal fluid away from black smoker activity (Fig. 2B). For simplicity, samples collected from short-fat tubeworm habitats are referred as “High Flow samples” and those collected from long-skinny tubeworm habitats as “Low Flow samples.” On shipboard, residual particulate detritus from the tubeworm grabs was collected from the bottom of the ROV bioboxes and immediately frozen at −80°C until further processing on shore.

### Carbon and nitrogen contents

A few grams of wet particular detritus from the three paired samples collected at S&M and Grotto vents, free of visible faunal organisms, were dried at 60°C for 24 h. After grinding to a fine powder, 30–80 mg of dry particular detritus were sent to the UC Davis Stable Isotope Facility (Davis, CA; http://stableisotopefacility.ucdavis.edu) for carbon and nitrogen analysis.

### DNA extraction and 454 tag sequencing

The eight samples were prepared for DNA extraction by sorting out the meiofauna using a Leica MZ16 stereomicroscope (Meyer Instruments, Houston, TX) in order to minimize eukaryotic DNA contamination. DNA was extracted and purified as described in Forget et al. (2010) and quantified using a Nanodrop ND-1000 spectrophotometer (Nano-Drop Technologies, Wilmington, DE). From this purified DNA, 10 μL of each sample, with a concentration of ∼10 ng/μL of DNA or higher, was sent to the Research and Testing Laboratory (Lubbock, TX), where the primers Gray28F 5'TTTGATCNTGGCTCAG-3' and Gray519r 5' GTNTTACNGCGGCKGCTG-3' were used to generate PCR amplicons of the bacterial small subunit (SSU) rRNA hypervariable regions V1–V3. Sequencing was carried out with a Roche 454 FLX instrument using the Research and Testing Laboratory protocols (http://www.researchandtesting.com)

### Pyrosequencing read analysis

In order to obtain high-quality sequences for statistical analysis, the original reads were passed through quality filters to reduce the error rate (Huse et al. 2007) using the software mothur v.1.22.1 (Schloss et al. 2009). Sequences with an average quality score below 25, containing ambiguous bases (N), shorter than 200 base pairs or not perfectly matching the forward primer or the barcode at the beginning of the read were eliminated. After trimming the primers and barcodes, potential chimeras were identified (and subsequently eliminated) using the program UCHIME (Edgar et al. 2011). The remaining sequences were aligned and classified using a SILVA reference database (Pruesse 2007) provided by mothur for bacterial sequences. A distance matrix was generated with mothur, and sequences having ≥97% similarity were treated as a same operational taxonomic unit (OTU).

### Statistical analysis of pyrosequencing reads

All of our analyses were performed using the software mothur and were based on the distance matrix and OTU definition mentioned above. Bacterial diversity within each of the eight samples was estimated with rarefaction analysis, coverage values, Chao1 richness, and Shannon diversity indices. Comparison of the structure and composition of the communities between samples were performed using a hierarchical clustering analysis and a nonmetric multidimensional scaling (NMDS) analysis with the Morisita–Horn calculator of dissimilarity (Horn 1966). The significance of the hierarchical clustering was tested using the weighted UniFrac algorithm (Lozupone and Knight 2005). We used an analysis of molecular variance (AMOVA) to test the significance of the spatial separation between High Flow and Low Flow samples in the NMDS plot, and an analysis of homogeneity of molecular variance (HOMOVA) to test the homogeneity of the bacterial communities between High Flow and Low Flow habitats. The command “metastats,” based on the program designed by White et al. (2009), was used to determine which OTUs were (significantly) differentially represented in the High Flow and Low Flow libraries.

### Sanger sequencing

Approximately, 10 ng/μL of DNA was used to amplify SSU rRNA gene sequences with the universal bacterial primers 8F (5'-AGA GTT TGA TCC TGG CTC AG-3') and 1492R (5'-GTT TAC CTT GTT ACG ACT T-3'). A range of MgCl_2_ concentrations and annealing temperatures were tested and the most stringent combinations with sufficient yield in the target size range were used. The final reaction mixture used (20 μL) contained 1 μL template DNA, 1.875 mmol/L MgCl_2_, 0.8 mmol/L deoxynucleoside triphosphates, 0.25 μmol/L (each) primer, 1X PCR buffer (Invitrogen, Valencia, CA), and 1 U of *Taq* DNA polymerase (Invitrogen). A first initialization step of 2 min at 94°C was followed by 26–30 cycles of denaturation at 94°C for 30 sec, annealing at 58°C for 45 sec, and extension at 72°C for 2 min. The final extension step was performed at 72°C for 10 min. The number of cycles was optimized for each sample in order to get a product concentration still in the exponential phase, as visualized on 1.2% (w/v) agarose gels stained with SYBR Safe (Invitrogen). Products of four parallel PCRs were combined, purified and cloned, and white colonies were randomly chosen and screened for inserts by PCR reaction using the vector primers M13F and M13R as described in Forget et al. (2010). Sequencing of the inserts was completed at the High-Throughput Genomics Unit (Seattle, WA) using the PCR primers 8F and 1492R, and 515F (5'-GTG CCA AGC MGC CGC GGT AA-3'), 519R (5'-GWA TTA CCG CGG CKG CTG-3'), 926F (5'-AAA CTY AAA KGA ATT GAC GG-3'), and 907R (5'-CCG TCA ATT CMT TTR AGT TT-3').

Repeated attempts to amplify Archaea with universal archaeal SSU primers ARCH-8F (5'-TCCGGTTGATCCTGCC-3') and ARCH-1492R (5'GGCTACCTTGTTACGACTT-3') yielded no product for any sample.

### Sanger sequence analysis

The SSU rRNA sequences were assembled and checked manually for errors using Sequencher v4.7 (Gene Codes Corporation, Ann Arbor, MI). The sequence data set was screened for potential chimeric structures using the Bellerophon server (Huber et al. 2004), available through the Greengenes website (DeSantis et al. 2006). Putative chimeras were further investigated with the program Pintail (Ashelford et al. 2005) by comparing each of them with closely related sequences recovered from the online tool BLAST (available through the National Centre for Biotechnology Information website). The sequences from each library were aligned independently using CLUSTAL W v2 (Larkin et al. 2007) and clones having 97% sequence similarity or higher were treated as the same OTU using DOTUR (Schloss and Handelsman 2005), based on the distance matrices obtained with the PHYLIP software package v3.68 (Felsenstein 2005). The phylogenetic affiliation of each sequence was assigned using the Classifier tool available through the Ribosomal Database Project (RDP) (Wang et al. 2007).

### Statistical analysis of Sanger sequences

The analyses described previously for the pyrosequencing data were also performed on the Sanger sequences. However, as the amount of information obtained from the first method provided more power to statistical tests, only the results from the phylogenetic affiliation of the sequences and the “metastats” command are discussed in this article.

### Nucleotide sequence accession numbers

The original pyrosequencing reads as well as their quality files were submitted to NCBI's Sequence Read Archive (SRA) and the project was assigned the accession number SRA056333. The SSU rDNA sequences representing unique OTUs have been submitted to the GenBank database and assigned the accession numbers JN662022 to JN662309.

## Results

### Carbon and nitrogen analysis

Three of the four paired samples were analyzed for carbon and nitrogen contents, while no more detritus was available for the two samples from Clam Bed. Total carbon and nitrogen concentrations were consistently higher in the Low Flow sample at each site (Table 2). In sample LF8GH2b, these concentrations were an order of magnitude higher than in the other samples, which could suggest the presence of tubeworm or meiofauna residue in the sample. The carbon/nitrogen molar ratios were also consistently higher in Low Flow samples.

**Table 2:** Carbon and nitrogen contents and molar ratio

Sample ID	%C	%N	C/N molar ratio
HF8SMb	0.28	0.06	5.56
LF8SMb	1.05	0.20	6.30
HF8GH1b	1.64	0.34	5.62
LF8GH1b	2.53	0.45	6.54
HF8GH2b	1.02	0.22	5.41
LF8GH2b	12.29	1.48	9.69

### Composition of sequence libraries

For both the Sanger and 454 tag sequencing approaches, a total of eight bacterial sequence libraries were constructed from the four paired samples. Several attempts to amplify archaeal SSU rRNA proved unsuccessful, which was also the case for other studies of microbial communities from diffuse hydrothermal environments (Corre et al. 2001; Alain et al. 2002, 2004; Forget et al. 2010). Similar efforts using samples from more severe hydrothermal conditions successfully amplified Archaea (Pagé et al. 2004; Byrne et al. 2009; Opatkiewicz et al. 2009). In this study, measured temperatures at sampling sites did not exceed 30°C.

#### 454 tag sequencing-based survey

**Figure 3:**
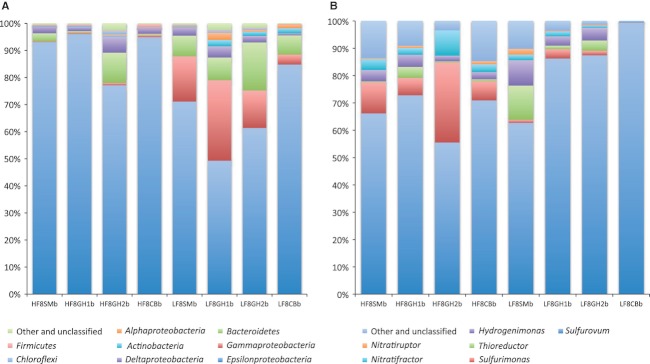
Relative abundance of (A) the major taxonomic groups and (B) the genera detected within the class *Epsilonproteobacteria* in the eight clone libraries constructed from 454 tag sequencing. Genera that did not reach 1% of the relative abundance of at least one library and unidentified operational taxonomic units (OTU)s were grouped under the category “Other and Unclassified”.

A total of 187,935 sequences covering the V1–V3 region of the bacterial 16S gene were obtained through 454 tag sequencing of our eight samples, 140,019 of which passed the quality filters described in the Experimental Procedures. This conservative approach reduced the size of our data set by ∼25%, similar to the results obtained by Sogin et al. (2006). The number of unique tags varied between 927 and 4563 per sample, and the number of OTUs varied between 364 and 1600 per sample (Table S1). These OTUs were affiliated with 29 different phyla of the bacterial domain, and 15 OTUs with <80% similarity to any cultured organism could not be confidently classified. The *Proteobacteria* dominated all libraries, accounting for over 80% of the total number of sequences per library, and up to 98.3% in the case of the High Flow sample HF8GH1b. Within this group, the *Epsilonproteobacteria* were the most abundant, representing from about 50% to 96% of the sequence libraries (Fig. 3A). Figure 3B shows the breakdown of this class at the genus level. The genus *Sulfurovum* was dominant in all libraries and accounted for 99.4% of the total number of *Epsilonproteobacteria* in the Low Flow sample LF8CBb, while the genus *Sulfurimonas* was more abundant in High Flow samples. The genera *Thioreductor*, *Hydrogenimonas*, *Nitratifractor*, and *Nitratiruptor* were also relatively abundant (Fig. 3B). The category “Other and Unclassified” representing <1% of the total *Epsilonproteobacteria* in any library, included metabolically diverse genera such as *Sulfurospirillum*, *Arcobacter*, *Wolinella*, *Campylobacter*, *Caminibacter,* and *Nautilia*.

The *Gammaproteobacteria*, mostly detected in the Low Flow samples, was the second most abundant class within the *Proteobacteria* (Fig. 3A). Sequences within this group were highly diverse, with over 60 different genera detected, most of which represented <1% of the total number of sequences (Table S2). Unclassified sequences accounted for 43% of the *Gammaproteobacteria*, but this number varied between libraries and reached 98.3% in the case of the High Flow sample HF8CBb. The most abundant genera that could be confidently identified were *Leucothrix*, *Endozoicomonas,* and *Methylosarcina*. *Thiohalophilus* was another relatively abundant genus, followed by *Dasania*, which accounted by itself for more than 50% of the gammaproteobacterial sequences detected in the Low Flow sample LF8CBb, and *Ectothiorhodosinus*, representing 1.4% of the total number of sequences.

The *Deltaproteobacteria*, which represented 2.6% of the total number of sequences, were also very diversified, with over 50 different genera detected (Table S3). Abundant genera included *Desulfobulbus*, *Desulfocapsa*, *Desulfonema*, *Desulfuromusa*, *Desulforhopalus,* and *Desulfoluna*. A few *Alpha-* and *Betaproteobacteria* were also detected (Tables S4 and S5).

The *Bacteroidetes* was the second most abundant phylum, followed by the *Actinobacteria*, the *Chloroflexi*, and the *Firmicutes* (Fig. 3A). The other phyla, including the *Acidobacteria*, *Lentisphaerae*, *OD1, Cyanobacteria, Deinococcus–Thermus, Chlorobi, Spirochaetes, Synergistetes, Tenericutes, Fusobacteria, WS3, SR1, Chlamydia, TM7, Gemmatimonadetes, Thermodesulfobacteria, Nitrospira, Planctomycetes, Verrucomicrobia,* and *Deferribacteres*, accounted for <1% of the relative abundance of any library and were grouped under the category “Other and Unclassified” in Figure 3A.

#### Sanger sequencing-based survey

**Figure 4:**
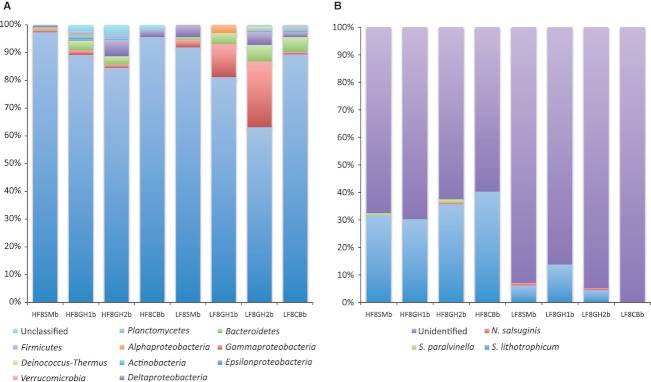
Relative abundance of (A) the phyla (the phylum *Proteobacteria*, which dominated all clone libraries, was divided into the four classes detected) and (B) species confidently identified within the class *Epsilonproteobacteria* in the eight clone libraries constructed from Sanger sequencing.

For the clone libraries constructed from Sanger sequencing, the number of clones sequenced per sample varied between 101 and 175, for a total of 1045 partial sequences. In each library, sequences that had 97% or greater similarity were considered as unique OTUs, and a representative of each OTU was fully sequenced. The number of OTUs detected per library varied between 18 and 51, for a total of 288. These OTUs that were affiliated with seven different phyla were also detected with 454 tag sequencing: the *Proteobacteria*, *Bacteroidetes*, *Actinobacteria, Planctomycetes*, *Verrucomicrobia, Deinococcus–Thermus,* and *Firmicutes* (Fig. 4A). Thirteen OTUs had <80% similarity to any cultured organism and could not be classified.

The general patterns of relative abundances of the most common groups were very similar to those observed in the pyrosequencing results (Figs. 3A, 4A). The *Proteobacteria* largely dominated the clone libraries, representing ∼95% of the clones sequenced. Within this group, the *Epsilonproteobacteria* were the most abundant group, representing 63–97% of the clone libraries (Fig. 4A). The *Gammaproteobacteria*, the second most abundant group, were mainly found in the Low Flow samples. The *Bacteroidetes*, *Deltaproteobacteria*, *Actinobacteria,* and *Alphaproteobacteria* followed in terms of relative abundance, similar to results obtained from pyrosequencing (Figs. 3A, 4A). The fully sequenced 16S gene allowed more precision when assigning phylogenetic affiliation; OTUs could be compared with previously published sequences and those with at least 97% similarity to cultured organisms could be identified at the species level. As only OTUs belonging to the *Epsilonproteobacteria* could be assigned to a species, Figure 4B shows the relative abundance of the identified species within this class. Between 32% and 42% of the *Epsilonproteobacteria* from the High Flow samples were affiliated with *Sulfurovum lithotrophicum*, while this species represented only a small proportion of the Low Flow libraries and was not detected in LF8CBb. A few OTUs from High Flow and Low Flow samples were affiliated with *Nitratifractor salsuginis*, while *Sulfurimonas paralvinellae* was detected only in High Flow samples (Fig. 4B).

Most epsilonproteobacterial OTUs could not be classified to the species level, and OTUs with <95% similarity to any cultured organism could not be assigned a genus. Their closest relatives in the GenBank database were environmental clones collected from hydrothermal vent chimneys, biofilms, and sediments, or uncultured symbionts of hydrothermal fauna such as the polychaetes *Alvinella pompejana*, *Paralvinella palmiformis* and *Riftia pachyptila*, the alvinocarid shrimp *Rimicaris exoculata*, and the recently discovered decapod *Kiwa hirsuta*.

A few gammaproteobacterial OTUs could be affiliated to the strictly aerobic genera *Granulosicoccus*, *Leucothrix,* and *Methylobacter*. However, most of them could not be confidently assigned a genus, but were closely related to environmental clones collected from hydrothermal vent systems and permanently cold marine sediments. The deltaproteobacterial genera *Desulfobulbus* and *Desulfocapsa* were detected. None of the *Alphaproteobacteria* detected could be confidently assigned a genus (data not shown).

Within the *Bacteroidetes*, the genus *Actibacter* was identified, but most of the phylotypes could not be assigned a genus. However, they were closely related to clones collected from hydrothermal vent environments, cold seeps, and permanently cold marine sediments. The OTUs detected in the *Actinobacteria*, *Planctomycetes*, *Verrucomicrobia*, *Deinococcus–Thermus,* and *Firmicutes* were not similar enough to any cultured organism to be identified at the genus level, but they were closely related to clones collected from deep-sea sediments, seafloor lavas, and polychaetes mucus and tubes.

### Diversity of the bacterial communities

**Figure 5:**
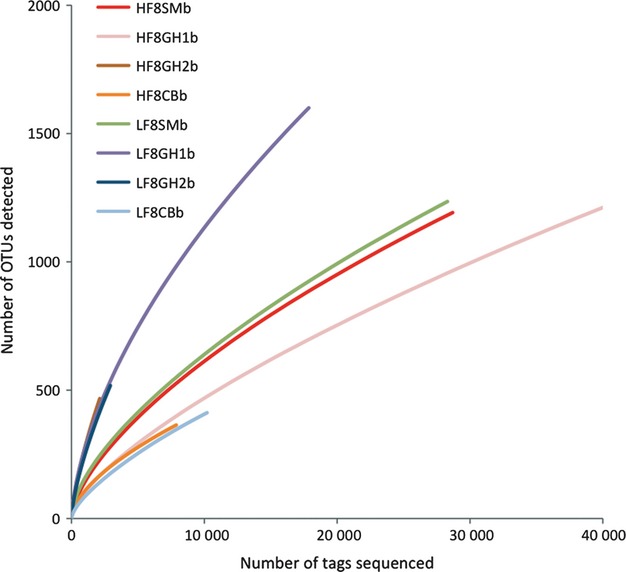
Rarefaction analysis of the libraries created from 454 tag sequencing showing the richness of each clone library at the 97% similarity level.

The tag sequencing data were used for diversity analysis as this method yielded considerably more information than with the Sanger sequencing. First, because the number of tags obtained differed among samples, rarefaction analyses, relating the number of OTUs detected to the sequencing effort, were used to compare the richness of the sequence libraries (Fig. 5). The samples from the Grotto vent site, except for HF8GHb1, had the highest richness, while the samples from Clam Bed had the lowest. High coverage values indicate that most of the bacterial diversity was encompassed by our sequencing effort (Table 3). The samples from Grotto whose rarefaction curves showed the highest richness had the lowest coverage scores. However, coverage, as well as the Chao1 index, is highly dependent on the sequencing effort, which explains the lower values for the paired samples from Hotspot 2 at Grotto vent, HF8GH2b and LF8GH2b (see Table S1, for total number of reads). The paired samples from Clam Bed, HF8CBb, and LF8CBb, also had a lower Chao1 richness index; while the number of tags sequenced for these samples was more than three times greater than the samples from Hotspot 2 at Grotto, and the number of OTUs represented by only one sequence (singletons) was lower, decreasing the Chao1 index value (Table 3). The Shannon–Wiener diversity index, which takes into account the richness and the evenness of the community, confirmed the lower diversity of the samples from Clam Bed. HF8GH1b had a high Chao1 index value, but also showed a lower Shannon–Wiener value, suggesting an uneven community.

**Table 3:** Diversity analyses of the clone libraries constructed from 454 tag sequencing

Sample ID	Coverage (%)	Chao1 richness index^1^	Number of singletons	Shannon–Wiener diversity index^1^
HF8SMb	97.4	3025 (2664–3474)	742	3.21 (3.18–3.24)
HF8GH1b	98.0	3581 (3140–4125)	850	2.84 (2.82–2.86)
HF8GH2b	85.0	1299 (1056–1618)	320	4.06 (3.96–4.16)
HF8CBb	97.3	869 (703–1115)	216	2.86 (2.81–2.91)
LF8SMb	97.3	3119 (2755–3568)	772	4.06 (4.04–4.08)
LF8GH1b	94.7	3725 (3357–4171)	943	4.97 (4.94–5.00)
LF8GH2b	87.8	1554 (1264–1956)	356	4.40 (4.32–4.47)
LF8CBb	97.3	1031 (847–1292)	273	2.19 (2.15–2.23)

1 Numbers in parenthesis indicate the 95% confidence intervals.

### Comparison of the bacterial communities

**Figure 6:**
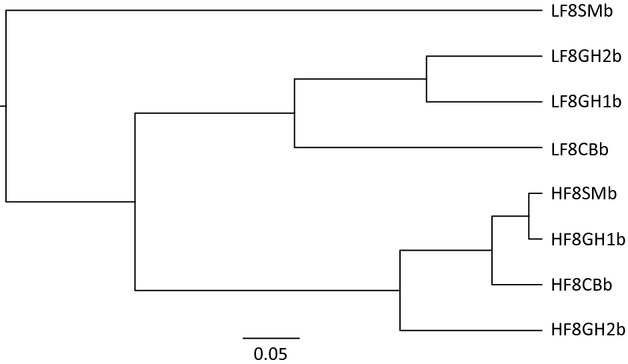
Hierarchical clustering analysis showing the similarity of the libraries constructed from 454 tag sequencing to each other using the Morisita–Horn calculator of dissimilarity. The scale bar represents the estimated divergence between the libraries.

**Figure 7:**
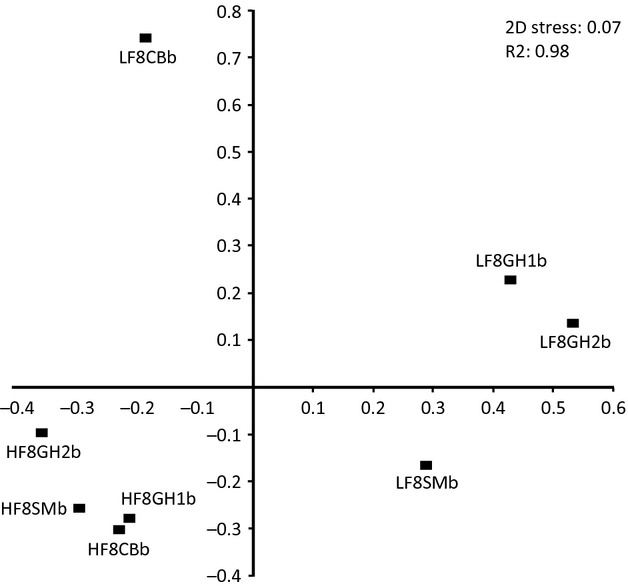
Nonmetric multidimensional scaling (NMDS) 2D similarity plot showing the distance between the libraries constructed from 454 tag sequencing based on the Morisita–Horn calculator of dissimilarity.

Two different visualization tools were used to compare the composition and structure of the bacterial communities between the libraries obtained from tag 454 sequencing. The tree constructed from the hierarchical clustering analysis showed all High Flow samples to be more similar to each other than to any other sample (Fig. 6). Three samples from the Low Flow habitats also grouped together, but sample LF8CBb from the Clam Bed Low Flow habitat did not cluster with any other sample, suggesting a unique bacterial community. A weighted UniFrac analysis, which tests the probability that the communities have the same structure by chance, showed that the separation of High Flow and Low Flow samples in the tree was highly significant (*P* < 0.001). The NMDS ordination plot corroborated the pattern observed with the hierarchical clustering analysis, showing all High Flow samples and three of the Low Flow samples grouped together (Fig. 7). The location of sample LF8CBb on the plot indicates that the bacterial community detected in this sample is distinct from the others. The AMOVA confirmed the significance of the spatial separation between High Flow and Low Flow samples with a *P* value <0.001. The plot also revealed shorter distances between the High Flow samples compared with the Low Flow samples, suggesting a greater diversity among the latter. The HOMOVA indicated that the diversity of the bacterial communities within each habitat was not homogeneous (*P* < 0.001). However, when sample LF8CBb was excluded from the HOMOVA analysis, no significant difference was observed between High Flow and Low Flow samples.

**Figure 8:**
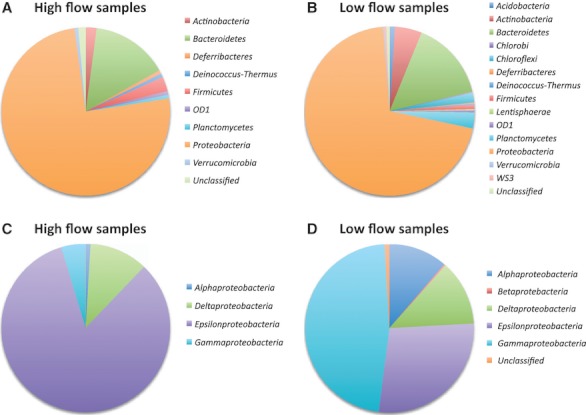
Relative abundance of the significantly differently represented operational taxonomic units (OTU)s (*P* < 0.05) in (A) High Flow phyla, (B) Low Flow phyla, (C) High Flow *Proteobacteria* and (D) Low Flow *Proteobacteria*.

The “metastats” function in mothur allows the identification of sequences that are differently represented between populations (*P* < 0.05). We found that a total of 142 OTUs belonging to at least 53 genera were differently represented among the High Flow samples, compared with 487 differently represented OTUs belonging to at least 167 genera in the Low Flow samples, respectively, accounting for 4.3% and 12.9% of the total number of OTUs in each habitat. A higher number of phyla were detected in Low Flow samples, but in both habitats, most differently represented OTUs belonged to the *Proteobacteria* and the *Bacteroidetes* (Fig. 8A and B). Within the *Proteobacteria*, differently represented OTUs in High Flow samples were mostly affiliated with the *Epsilonproteobacteria*, followed by a few *Deltaproteobacteria*, five *Gammaproteobacteria,* and one *Alphaproteobacteria* (Fig. 8C), while in Low Flow samples, most differently represented OTUs were affiliated with the *Gammaproteobacteria*, followed by the *Epsilonproteobacteria*, the *Deltaproteobacteria*, the *Alphaproteobacteria*, one *Betaproteobacteria,* and three unclassified *Proteobacteria* (Fig. 8D). A closer look at the genus level revealed that High Flow and Low Flow samples were colonized by different species that belong mostly to the *Sulfurovum*, *Sulfurimonas*, *Hydrogenimonas,* and *Nitratifractor* within the *Epsilonproteobacteria*, and to the genus *Desulfobulbus* within the *Deltaproteobacteria*. Within the *Gammaproteobacteria*, the most abundant genera detected in the Low Flow libraries were *Leucothrix* and *Endozoicomonas*. Other relatively abundant phyla differently represented between habitats included the *Actinobacteria*, the *Planctomycetes,* and the *Chloroflexi,* mostly detected in Low Flow libraries. The same analysis performed on the clone libraries constructed from Sanger sequencing showed *S. lithotrophicum* to be more represented in High Flow samples (*P* < 0.001), while the other OTUs with significantly different representation between habitats had <97% sequence similarity to any cultured organism and could not be classified to the species level.

## Discussion

Our survey combined conventional Sanger sequencing with 454 tag sequencing; the first approach provided more precise information on the taxonomy of the sequences, while the second allowed rapid generation of large numbers of sequences without the bias introduced by the cloning step, thereby enabling a deeper survey and better coverage of bacterial diversity and the detection of rare species. Furthermore, while only semi-quantitative, 454 tag sequencing provides a more accurate estimate of the relative abundance of OTUs in the microbial communities (Sogin et al. 2006).

Neither of the two molecular approaches used in this study yielded any archaeal sequences. Alain et al. (2004) pointed out that while mesophilic Archaea are known to be ubiquitous, only thermophilic and hyperthermophilic strains have been detected in hydrothermal vent environments. Furthermore, these strains were mostly anaerobic or microaerophilic organisms, while diffuse flow habitats are zones of mixing between hydrothermal fluids and well-oxygenated background seawater (Alain et al. 2004). Roussel et al. (2011) used nested PCR to improve yield of 16S rRNA gene PCR product from samples from three hydrothermal vent sites on the Mid-Atlantic Ridge. Even with this approach, results were mixed with not all samples yielding sufficient PCR product for cloning.

The use of 454 tag sequencing technology allowed the detection of 29 different bacterial phyla, more than four times the number of phyla detected by Sanger sequencing. The substantially greater information yield from the 454 tag sequencing-based survey increased the power of the statistical analyses, thereby reducing the probability of type II errors, the failure to reject a false null hypothesis, that could have prevented detection of patterns related to habitat type. Yet, the relative abundance of the major taxonomic groups detected in each sample was comparable with the Sanger sequencing survey. Unsurprisingly, the *Epsilonproteobacteria*, which play a major role in the cycling of nitrogen and sulfur, were dominant in all libraries. This has been observed in numerous microbial diversity surveys of hydrothermal habitats, including venting fluids (Huber et al. 2010), mats covering surfaces in the vicinity of vents (Zhou et al. 2009; Flores et al. 2011; Lanzen et al. 2011), or in association with vent fauna (Alain et al. 2002, 2004; Lopez-Garcia et al. 2002). Both of our surveys found the facultative anaerobic mesophilic sulfur-oxidizing genus *Sulfurovum* to be dominant in all samples (Inagaki et al. 2004). The genus *Sulfurimonas*, whose members are also mesophilic chemolithoautotrophs using similar electron donors but in aerobic conditions (Inagaki et al. 2003), was mostly represented in High Flow samples. Other relatively abundant Epsilonproteobacterial genera included the mesophilic to thermophilic and strictly chemolithoautotrophic *Thioreductor*, *Hydrogenimonas*, *Nitratifractor*, and *Nitratiruptor*, all using molecular hydrogen as electron source in anaerobic to microaerophilic habitats (Takai et al. 2004; Nakagawa et al. 2005a,b). These genera have in common metabolisms well adapted to vent conditions and are therefore frequently detected in hydrothermal environments (Campbell et al. 2006).

Both surveys also agreed on the high relative abundance of the class *Gammaproteobacteria* in Low Flow libraries, but a large proportion of the OTUs could not be classified to the genus level, and the majority of the identified genera accounted for <1% of the sequences. However, the Sanger survey showed that most of these OTUs were closely related to uncultured bacteria collected from vent chimneys or living in association with vent fauna that were also sampled from the Endeavour Segment of the Juan de Fuca Ridge, and described, respectively, as methanotrophs and decomposers involve in the degradation of organic debris (Alain et al. 2002; Wang et al. 2009). These descriptions are consistent with the most abundant genera that were confidently identified in this study, such as *Leucothrix*, *Endozoicomonas, Methylosarcina,* and *Dasania* from the pyrosequencing survey and *Granulosicoccus* and *Methylobacter* from the Sanger sequencing survey (Harold and Stanier 1955; Bowman et al. 1993; Wise et al. 2001; Kurahashi and Yokota 2007; Lee et al. 2007a,b). The occurrence of methanotrophs in our samples suggests the presence of CH_4_ in hydrothermal fluids emitted at these sites, confirmed by the elevated concentrations measured by de Angelis et al. (1993). Free-living methanotrophs have been reported from many hydrothermal sites (Takai et al. 2006) and are also part of the epibiotic fauna found on the Mid-Atlantic shrimp *R. exoculata* (Zbinden et al. 2008; Guri et al. 2012). Interestingly, only two relatively abundant *Gammaproteobactera* genera found in our samples, *Ectothiorhodosinus* and *Thiohalophilus,* have been described as sulfur oxidizers (Gorlenko et al. 2004; Sorokin et al. 2007). The dominance of *Epsilonproteobacteria* at all sites would suggest that metabolisms using sulfur compounds as electron donors should also be common among the metabolically diverse *Gammaproteobacteria*. It is possible that diffuse flow conditions at these sites provided sulfur-metabolizing *Epsilonproteobacteria* with a competitive advantage over their *Gammaproteobacteria* counterparts.

The most abundant genera detected within the class *Deltaproteobacteria* included strictly anaerobic and heterotrophic genera, such as *Desulfobulbus*, *Desulfocapsa*, and *Desulforhopalus* detected by both methods, and *Desulfonema*, *Desulfuromusa*, and *Desulfoluna* detected only by 454 tag sequencing (Widdel and Pfennig 1982; Widdel et al. 1983; Liesack and Finster 1994; Isaksen and Teske 1996; Janssen et al. 1996; Suzuki et al. 2008). Members of these taxonomic groups are sulfur reducers, and their presence along with sulfur oxidizers from the *Epsilonproteobacteria* and the *Gammaproteobacteria* classes might suggest internal sulfur cycling within these habitats, as proposed by Forget et al. (2010) and Lanzen et al. (2011) in studies of other low-temperature hydrothermal environments.

The OTUs that belonged to the other detected major phyla, including the *Bacteroidetes*, *Actinobacteria*, *Chloroflexi,* and *Firmicutes*, were all members of heterotroph genera, and most likely involved in the decomposition of organic matter. The minor groups were metabolically diverse, and a relatively large proportion could not be classified to the genus level, but identified genera were also heterotrophs.

### Diversity indices and community composition

Our results showed a significant relationship between microbial community composition and habitat type, suggesting a predictable pattern in High Flow and Low Flow environments. In the tree constructed from the cluster analysis, all libraries sampled from a same habitat at different vent sites, except one, grouped together, and the spatial separation of the High Flow and Low Flow libraries in the NMDS ordination plot was highly significant. The Low Flow library from Clam Bed did not cluster with any other habitat, indicating that the microbial communities from this Low Flow site were distinct from all other communities investigated. Furthermore, the spatial distribution of the Low Flow libraries on the NMDS plot, more widely spread along both axes, suggested that the overall diversity within this habitat, when considering all samples, was higher than that of the High Flow habitat. The distinct composition of the Low Flow sample from Clam Bed was primarily responsible for the apparently higher diversity in the Low Flow habitat. Removing the Clam Bed Low Flow library from the NMDS analysis resulted in there being no significant difference between High Flow and Low Flow libraries in terms of overall diversity. The High Flow library collected from the same site did not diverge from the other libraries in terms of composition. However, both samples from Clam Bed had significantly lower Chao1 richness and Shannon–Wiener diversity indices compared to most other samples, and high coverage values, suggesting that the microbial communities at this vent site were less complex than the other sites.

This result could be explained by a difference in sources of energy available between sites, especially between High Flow habitats, possibly limiting the diversity of the bacterial taxonomic groups adapted to the environmental conditions at Clam Bed. The observation of chemical gradients in the composition of hydrothermal fluids observed along the Endeavour Segment (Butterfield et al. 1994), together with the location of the sites (while the three other sites sampled are all within 150 m of each other, Clam Bed is ∼1750 m away from Grotto and Smoke & Mirrors) supports this explanation. This is also coherent with the results obtained by Flores et al. (2011) showing the composition of the microbial communities to be more similar within vent fields than between them, suggesting that the effect of distinct environmental conditions between vent fields is more important than any other variable within a vent field. However, the chemical components of fluids in Low Flow habitats are very diluted. Therefore, the difference in the composition of the fluids between sites is unlikely to explain the lower diversity in the Low Flow sample from Clam Bed. Also, the composition of the microbial communities showed no statistically significant difference within or between vent field relationships (data not shown). This was also the case in a recent study of the patterns of distribution of *Epsilonproteobacteria* in hydrothermal fluids along the Mariana Arc, where the communities from individual vents seemed to be unique (Huber et al. 2010). These authors suggested that the high diversity of chemical conditions in hydrothermal environments might explain the distinctness of the epsilonproteobacterial populations.

Another fact to take into account is the temporal stability of the sampled habitats. Except for the Low Flow sample from Clam Bed, all samples were collected directly on active sulfide edifices, with the High Flow samples typically found adjacent to vigorous venting and the Low Flow samples further away, on the same edifice, where little or no shimmering flow was visible. These structures are known to be very unstable environments that rapidly evolve at small spatial scales, affecting the distribution and structure of the macrofaunal communities (Sarrazin et al. 1997, 2002). In contrast, the Low Flow sample from Clam Bed was collected from a basaltic substratum in an area where diffuse flow has supported communities of *R. piscesae* since at least 1991 (Reyes et al. 1995; Tivey et al. 1999; Durand et al. 2006). The stability of this site compared with the others may explain the development of a distinct microbial community.

In order to understand other features distinguishing the High Flow and Low Flow habitats, we explored the taxonomic affiliation of the OTUs that were differently represented between habitats. OTUs that were significantly more represented in our Low Flow libraries were over three times more abundant than in our High Flow libraries. This observation suggests that bacterial groups representing only a small proportion of High Flow communities might play a more important ecological role in Low Flow communities. Also, several species were significantly more abundant in the High Flow libraries than in the Low Flow libraries. These include mesophilic to thermophilic microaerophilic sulfur and hydrogen oxidizers from the class *Epsilonproteobacteria,* with one organism classified to the species level, *S. lithotrophicum*, as well as a few heterotrophs from the phylum *Bacteroidetes* and sulfur reducers from the *Deltaproteobacteria*. Different species from the same genera having similar metabolisms also proved to be more represented in Low Flow samples, but most of the OTUs significantly more abundant in this habitat belonged to heterotrophic groups, including methanotrophs of the class *Gammaproteobacteria*. Other relatively abundant heterotrophic genera from the phyla *Actinobacteria*, *Planctomycetes,* and *Chloroflexi* were also more represented in Low Flow libraries.

Differences in temperature, available energy for metabolism, and stability between High Flow and Low Flow habitats potentially explain their distinctive bacterial communities. Even though sulfur and hydrogen oxidizers dominate both types of habitat, the fact that some species were significantly more abundant in High Flow or Low Flow samples suggests that environmental conditions that control tubeworm morphotypes and the composition of their associated macrofauna can also shape the composition of free-living bacterial communities. Environmental shaping of hydrothermal vent microbial communities has also been observed in other studies (Schrenk et al. 2003; Byrne et al. 2009; Kato et al. 2010; Flores et al. 2011), although it is not usually considered in the broader ecological context of factors controlling the composition of vent faunal assemblages. The abundance of heterotrophic organisms in Low Flow libraries (vs. High Flow) indicates that detrital organic matter may be a more important source of energy and carbon for the microbial communities in this habitat. Perner et al. (2007) also point out the potential importance of heterotrophic metabolism in microbial assemblages at low-temperature vents, although their study did not include analysis of available organic matter. Our carbon and nitrogen analyses consistently showed High Flow sites to have fresher, less degraded detritus (lower C/N ratios), which should normally provide more energy for heterotrophic growth than the more refractory organic material (higher C/N ratios) sampled from the Low Flow sites. This is consistent with findings by Venkitachalam (2005) that both autotrophic and heterotrophic microbial enzyme activity were more intensive in detrital samples from High Flow tubeworm colonies than in samples from Low Flow sites. Despite the fact that Low Flow habitats represent lower energy environments (compared to High Flow sites), where the availability of reduced sulfur species is limited (Urcuyo et al. 2003) and fresh organic matter is scarce, there were no major differences in overall bacterial diversity between the two habitats. All conditions being equal, one would expect the higher energy habitat to support a greater level of bacterial diversity. One important difference between the High Flow and Low Flow habitats may be related to their relative stability and longevity. High Flow habitats have been observed to be short lived (Sarrazin et al. 1997; Tunnicliffe et al. 1997) while at least one of the Low Flow sites sampled here (Clam Bed) is known to have been active for at least two decades. The greater stability of the Low Flow sites may have therefore favored a diversification of both autotrophic and heterotrophic bacteria, despite the less favorable growth conditions.

Hydrothermal vents are extremely dynamic environments where physical and chemical conditions vary rapidly both in time and space, and this is known to affect the composition of the macrofaunal communities (Sarrazin et al. 1999, 2002). To date, logistic considerations have limited the use of replicate sampling in studies of the dynamics of vent faunal communities, thus limiting the scope of any interpretation of observed differences between samples. For similar practical reasons, replication and statistical comparisons have been even less common in comparative studies of hydrothermal vent microbial communities. As the field progresses from general descriptive studies to probing relationships between microbial diversity and habitat dynamics, replicate sampling and statistical comparisons will become essential to identifying trends and testing hypotheses. In this study, we have provided some examples of the insights that can be derived from a combination of systematic, replicated field collections, and molecular analysis of microbial diversity. Even if the small number of cultured microorganisms known from hydrothermal vent habitats, combined with the limitation of taxonomic assignment by 454 tag sequencing data prevented us from investigating which species defined the bacterial communities of High Flow and Low Flow habitats, our survey revealed interesting general characteristics about these communities, such as their high overall diversity and a probable relationship of composition with habitat type. Furthermore, results underline the ubiquity of *Epsilonproteobacteria* in hydrothermal vent environments, which were mainly related to sulfur- and hydrogen-oxidizing genera. Other approaches, such as metagenomic and proteomic analysis, could help to define more specifically the ecological role of the microorganisms detected by providing information on genes and enzymes specific to each habitat.

## Supporting Information

**Table S1.** 454 tag sequencing summary.

**Table S2.** Occurrence of the gammaproteobacterial genera within the eight sequence libraries. The genera in bold represented more than 1% of the total number of sequences.

**Table S3.** Occurrence of the deltaproteobacterial genera within the eight sequence libraries. The genera in bold represented more than 1% of the total number of sequences.

**Table S4.** Occurrence of the alphaproteobacterial genera within the eight sequence libraries.

**Table S5.** Occurrence of the betaproteobacterial genera within the eight sequence libraries.
